# Long non-coding RNAs and autophagy: dual drivers of Hepatocellular carcinoma progression

**DOI:** 10.1038/s41420-025-02667-7

**Published:** 2025-08-11

**Authors:** Himanshi Goyal, Jyotdeep Kaur

**Affiliations:** Department of Biochemistry, Post Graduate Institute of Medical Education and Research, Chandigarh, 160012 India

**Keywords:** Cancer prevention, Metastasis

## Abstract

Hepatocellular carcinoma (HCC), a leading cause of cancer-related mortality worldwide, is characterized by poor prognosis, high recurrence rates, and limited responsiveness to current therapies. Autophagy, a conserved catabolic pathway essential for cellular homeostasis, plays a paradoxical role in HCC, acting as a tumor suppressor during initiation but promoting survival and progression in advanced stages. Long non-coding RNAs (lncRNAs) have emerged as critical regulators of autophagy, influencing tumorigenesis, metastasis, and therapy resistance through mechanisms such as miRNA sponging, chromatin remodeling, and protein interactions. This review describes how autophagy contributes to HCC at different stages, outlines the dual functions of lncRNAs as oncogenic drivers or tumor suppressors, and illustrates their integration into key signaling networks of autophagy (e.g., PI3K/AKT/mTOR, AMPK, Beclin-1). LncRNAs have been shown to modulate drug resistance, including resistance to first-line agents, by altering autophagic flux and associated molecular pathways. We also explored emerging strategies for targeting the lncRNA–autophagy axis, such as siRNAs, antisense oligonucleotides, and CRISPR/Cas systems, that have shown promise in preclinical studies and may be adapted for HCC. Furthermore, autophagy-related lncRNAs hold potential as non-invasive diagnostic and prognostic biomarkers and as predictors of recurrence. Integrating multi-omics approaches to validate these candidates will be critical for translation into clinical practice. Collectively, this review highlights the lncRNA–autophagy network as a promising frontier of biomarker discovery for precision diagnostics and targets for innovative therapeutics. The regulatory role of lncRNAs in autophagy presents a paradigm shift, heralding new strategies for targeted treatment.

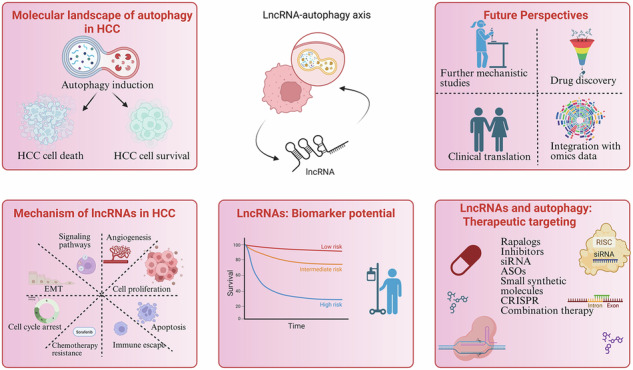

## Open Questions

- In what ways does the tumor microenvironment modulate autophagy during hepatocarcinogenesis?
- How can the context-dependent role of autophagy in HCC be therapeutically exploited?
- What challenges exist in targeting lncRNAs therapeutically, particularly in the context of modulating autophagy in HCC?
- How might lncRNA-based risk-stratification models improve current clinical decision-making in HCC?
- What ethical or safety concerns might arise with CRISPR or ASO-based therapeutic strategies targeting lncRNAs in HCC?
- How can multi-omics approaches be integrated to identify key lncRNA–autophagy axes in HCC?

## Introduction

Hepatocellular carcinoma (HCC) is one of the most common forms of liver cancer and poses a major global health concern [1]. It ranks among the top five causes of cancer-related mortality, with most patients facing poor prognosis and a dismal 5-year survival rate of less than 20% [2]. While standard treatment options- including surgical resection, local interventions, and systemic therapies, remain the cornerstone of HCC management across various clinical stages [3]. However, despite advancements in systemic treatments, the majority of patients exhibit low response rates and eventually succumb to the disease [4]. These challenges highlight an urgent need for new and more effective therapeutic strategies to improve clinical outcomes in HCC.

**Fig. 1:**
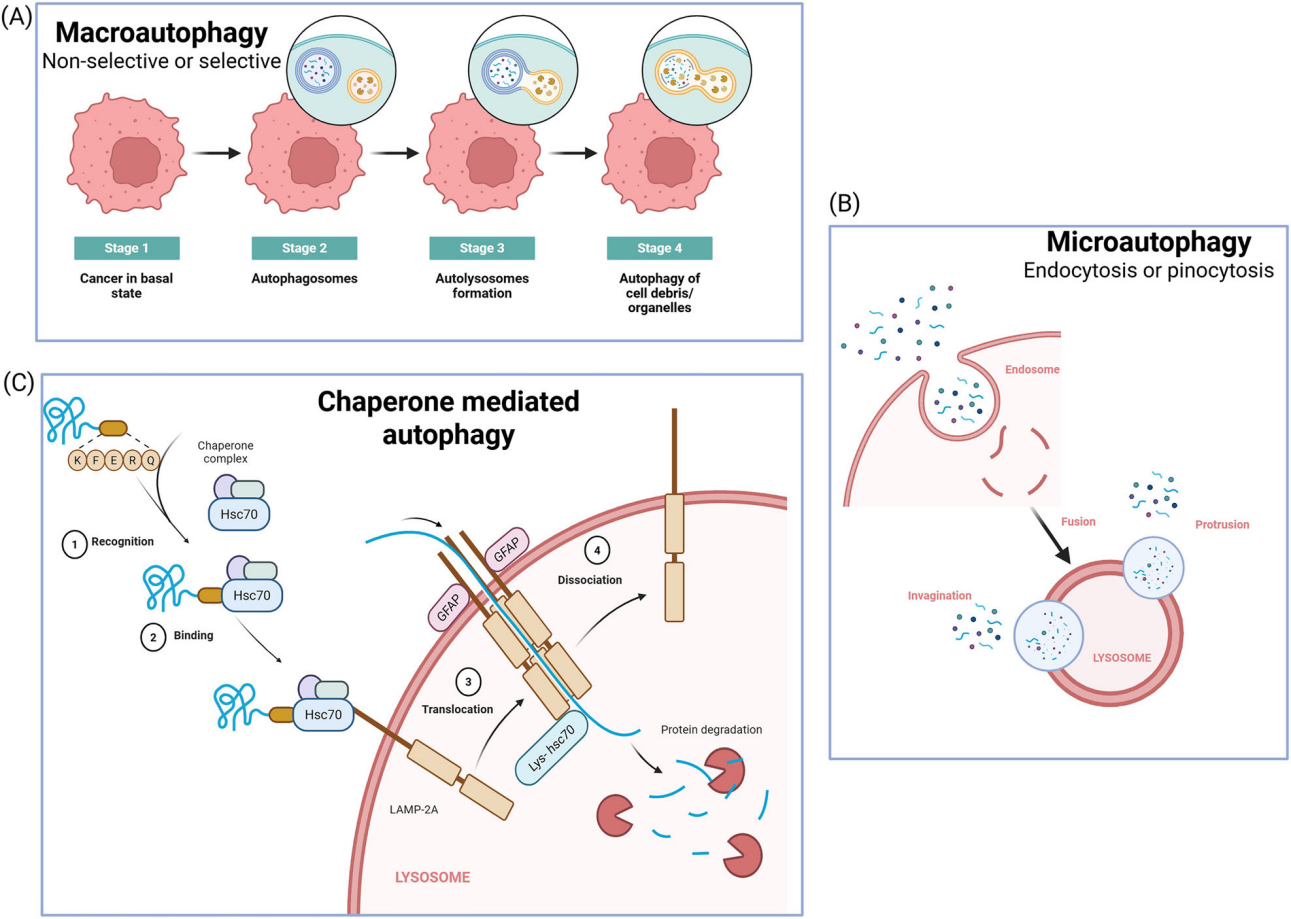
Three major types of autophagy: macroautophagy, microautophagy, and chaperone-mediated autophagy (CMA). **A** Macroautophagy-a process that can be selective or non-selective, involving the formation of autophagosomes that engulf cellular components. **B** Microautophagy- A lysosome-driven process where small portions of cytoplasmic components are engulfed by invagination, protrusion, or fusion of the lysosomal membrane. This process occurs via endocytosis or pinocytosis. **C** Chaperone-mediated autophagy- A highly selective pathway involving recognition of cytosolic proteins containing a specific KFERQ motif by Hsc70 chaperone; binding of the protein-chaperone complex to LAMP2A on the lysosomal membrane; translocation of the substrate protein into the lysosome, and dissociation and degradation of the protein inside the lysosome.

Under stressful conditions, tumor cells activate adaptive mechanisms like ER stress due to the increase in demand for protein biosynthesis, as reviewed in our own paper [5]. But the time and cellular state determine the activation of either the pro-adaptive state by inducing autophagy or activation of the cell death mechanisms, viz., apoptosis under chronic stress. The autophagy-lysosomal system, an integrated mechanism that combines the autophagy and lysosomal functions, plays a critical role in preserving cellular health and homeostasis. Autophagy is not merely the removal of waste or waste degradation but a highly regulated catabolic mechanism that targets a broad range of intracellular components—including misfolded proteins, damaged organelles (such as mitochondria and peroxisomes), lipid droplets, invading pathogens, and protein aggregates for lysosomal degradation and recycling. Autophagy exists in three primary forms: microautophagy, macroautophagy, and chaperone-mediated autophagy (CMA). Microautophagy involves the direct capture of the cytosolic components, which are sequestered through the invaginations at the lysosomal membrane whereas CMA is the selective form of autophagy, lysosomal membrane receptors such as LAMP2A (lysosomal-associated membrane protein 2A) recognize and translocate specific proteins marked with a KFERQ amino acid motif [6]. These proteins, complexed with chaperones such as HSPA8/HSC70 (heat shock protein family A Hsp70 member 8), are unfolded as they cross into the lysosomal lumen, where they undergo degradation [7]. In this article, we focused only on macroautophagy, commonly referred to as autophagy, characterized by the formation of double-membrane structures called autophagosomes (encapsulate the damaged organelles and proteins), that fuse with lysosomes leading to the formation of autolysosomes, which degrades the contents by lysosomal enzymes and causes the recycling of the broken products [8]. Figure 1 summarizes the three types of autophagy: macroautophagy, microautophagy, and chaperone-mediated autophagy.

Understanding the intricate relationship between autophagy and HCC could pave the way for targeted therapies that exploit this mechanism for improved patient outcomes. This review emphasizes the epigenetic modulation of autophagy by lncRNAs in HCC and discusses their potential, along with downstream miRNAs, as targets for novel therapeutic strategies.

## Molecular landscape of autophagy in HCC

### Mechanism of autophagy

Autophagy is a highly conserved evolutionary mechanism essential for protein and organelle turnover, playing a pivotal role in maintaining cellular quality {as reviewed in [9]}. This lysosome-dependent process primarily aims to supply nutrients and energy via the degradation of cytoplasmic contents. Emerging evidence also highlights its influence on cellular specialization, differentiation, and protein trafficking [6, 10]. The protective mechanism of autophagy is already linked with various cardiovascular diseases, neurodegenerative diseases, metabolic diseases, cancer, etc. [11–13]. However, autophagy acts as a double-edged sword in activating and inhibiting apoptosis, which depends upon the cellular state and the trigger for the autophagy activation, which prompts the cells to adapt to new conditions [14, 15]. The key steps of macroautophagy include: Initiation, nucleation, elongation and maturation, autophagosome formation, fusion with lysosome, followed by degradation and recycling. Autophagy is the process of degradation of waste from the cells characterized by the formation of the double-membrane autophagosomes, a process mediated by core molecular machinery which includes ULK1 complex, the class III PI3K complex, and the ATG8 conjugation system, all of which coordinate different stages of the autophagy cascade[16]. Two pivotal cellular energy sensors viz. mTOR and AMPK, tightly regulate autophagy initiation in response to environmental cues like nutrient availability and stress. Under the conditions of nutrient deprivation, AMPK activates ULK1 whereas inhibition of mTORC1 relieves its suppressive effect, as a result ULK1 phosphorylates downstream autophagy-related proteins and initiates production of autophagic vesicles [17]. The catalytic domain in the mTOR is quite similar to the PI3K [18]. mTOR complex includes:

- mTORC1: Composed of mTOR, Raptor, PRAS40, and mLST8. It is nutrient-sensitive and negatively regulates autophagy by phosphorylating ULK1 (at Ser757), which inhibits AMPK-induced activation of ULK1 [19].
- mTORC2: Composed of mTOR, Rictor, SIN1, and mLST8. It regulates cell survival, cytoskeletal remodeling, and AKT phosphorylation at Ser473, which indirectly inhibits autophagy via downstream activation of mTORC1 [20].

The PI3K complex, which includes VPS34 and Beclin-1, is essential for the nucleation of the phagophore- the initial membrane structure that gives rise to the autophagosome. Upon activation, the kinase JNK phosphorylates the pro-survival proteins BCL-2 and BIM, leading to the disruption of their inhibitory complexes with Beclin1. This phosphorylation event liberates Beclin1, allowing it to interact with VPS34, a class III PI3K. The resulting Beclin1–VPS34 complex catalyzes the production of phosphatidylinositol 3-phosphate (PI3P), which facilitates the nucleation and expansion of the autophagic membrane during early autophagosome formation {as reviewed in [21]}. Once nucleation occurs, the ATG8 system, consisting of the LC3 and GABARAP protein subfamilies, mediates the elongation and closure of the autophagosomal membrane. The ATG12–ATG5 conjugate interacts with ATG16L1, forming the multimeric ATG12–ATG5–ATG16L1 complex [22]. This complex assembles the phagophore membrane and plays a crucial role in the elongation and expansion of the autophagosomal membrane. Once formed, it localizes to the outer surface of the growing autophagic vesicle, where it facilitates the lipidation of ATG8 family proteins and supports autophagosome maturation. In the last step, which is the fusion with lysosomes, LC3 incorporation into autophagosomes involves a multistep enzymatic process. Initially, the cysteine protease ATG4 cleaves pro-LC3 to generate LC3-I, which exposes a C-terminal glycine residue [23]. LC3-II is subsequently integrated into the autophagosomal membrane, where it plays a key role in cargo recognition and autophagosome maturation. Following autophagosome completion, it fuses with a lysosome to form an autolysosome, enabling degradation of the enclosed cellular material.

### Role of autophagy as a tumor suppressor in early HCC

Under the physiological conditions, both macroautophagy and chaperone-mediated autophagy operate at basal levels, primarily facilitating the degradation of dysfunctional cellular components and thus maintaining liver homeostasis. During the early stages of hepatocarcinogenesis, macroautophagy is the first to respond to energy deprivation due to energy consumption by tumor cells. The tumor microenvironment, characterized by adverse conditions such as oxidative stress and metabolic imbalance, induces macroautophagy activation, when it acts as the tumor suppressor, eliminating damaged organelles, preventing inflammation, and reducing p62 accumulation, thereby maintaining cellular integrity under stress [24]. Studies by Sun et al. have shown that macroautophagy inhibition in animal models accelerates hepatocarcinogenesis at the dysplastic stage yet paradoxically suppresses tumor formation in later stages [25]. In addition, it has been observed that deletion of Beclin1 is related to the development of HBV-related HCC, and its levels have also been studied as the independent prognostic marker for HCC progression [26]. Not only this, the liver shows a high dependency on autophagy pathways to maintain homeostasis as evidenced by the increased malignancies due to the loss of ATG5 and ATG7 (key macroautophagy genes) [6]. This tumor-suppressive role is attributed to its ability to clear protein aggregates, mutated proteins, and damaged organelles. Failure in the process triggers oxidative stress, inflammation, and genomic instability [27]. Deficient macroautophagy in cancerous hepatocytes results in elevated oxidative stress and p62 accumulation, both of which play a critical role in HCC development and malignant transformation [28]. The suppression of macroautophagy impairs the lysosomal degradation of p62 and mutated proteins, leading to their accumulation and contributing to tumor progression [29]. Apart from this, chronic p62 accumulation drives the sustained activation of NF-κB, NRF2, and the mTOR pathway, which collectively promote tumor survival and progression [30]. While NF-κB and NRF2 mitigate inflammation and oxidative stress, mTOR signaling ensures sufficient energy availability for cancer cell growth [31].

### Role of autophagy as the tumor survival mechanism in advanced HCC

During the HCC progression, stress and metabolic pressure build-up, resulting in the upregulation of chaperone-mediated autophagy, acting as the compensatory mechanism. At this stage, macroautophagy declines, evidenced by the accumulation of p62, activating NRF2 and promoting mTOR signaling driving tumor cell proliferation and growth, independent of growth factors [28]. Long-term starvation and energy deficit conditions lead to the activation of CMA, which characterizes the tumor microenvironment. CMA activation under such conditions in liver cells leads to degradation of Beclin-1, thereby autophagy inhibition at the initiation stage and disrupting autophagosome-lysosome fusion {as reviewed by [32]}. Studies on the HCC nodules in the cirrhotic livers revealed the elevated expression of LAMP2A, a key CMA marker. LAMP2A is also recognized as an indicator of lysosome proliferation, facilitating enhanced cellular degradation and autophagic cargo recycling to support the heightened energy demands of cancer cells [33]. Under chronic HCV (Hepatitis C virus) infection which is the leading risk factor for HCC, persistent cellular stress activates the NRF2 pathway, a crucial antioxidant defense mechanism that promotes cell survival. Additionally, NRF2 functions as a transcription factor for key CMA components, including LAMP2A and HSC70, upregulating their expression and potentially activating CMA under severe stress conditions [34]. Lastly, it has been reported that mTORC2 which is the second complex of the mTOR pathway suppresses CMA activation during HCV infection via AKT kinase [35].

Altogether, autophagy plays a complex and dual role in HCC progression, acting as both a tumor suppressor and promoter depending upon the cellular context and associated regulatory pathways. For instance, Wnt and the Akt signaling pathways are the two major pathways linked to cell growth, differentiation, survival, and tumorigenesis and are related to the activation/inhibition of autophagy. In fact, tumor necrosis factor-α-induced protein 8 (TNFAIP8) leads to increased cell survival in HCC by blocking the AKT/mTOR pathway to induce autophagy and steatosis in liver cells [36]. In contrast to this, PNO1, an RNA-binding protein, is associated with the poor prognosis of HCC patients via the induction of autophagy and suppression of apoptosis through the MAPK signaling pathway [37]. Not only this, ZNF263, a key ER stress specific super- enhancer associated transcription factor, is significantly upregulated in HCC patients and enhances chemoresistance by promoting ER stress-related autophagy. This finding suggests a key role of autophagy as the tumor suppressor in early HCC, a pro-survival mechanism for tumor cells in advanced HCC along with the therapy resistance [38].

## Long non-coding RNAs in HCC

### LncRNAs as oncogenes and tumor suppressors

LncRNAs serve as critical regulators of HCC, playing pivotal roles in initiation, development, progression, and inhibition. A growing body of research has demonstrated that several lncRNAs function as oncogenes, driving tumorigenesis through diverse molecular mechanisms and acting as tumor suppressors, inhibiting tumor formation. One of the primary ways lncRNAs exert their effect is by acting as molecular sponges for microRNAs, a process known as the competing endogenous RNA (ceRNA) network. By binding to tumor-suppressive miRNAs, these lncRNAs prevent the degradation of oncogenic mRNA targets, thereby promoting the expression of genes that drive tumor growth and survival. Additionally, some oncogenic lncRNAs participate in chromatin remodeling and transcriptional regulation, either by interacting with chromatin-modifying complexes such as PRC2 (Polycomb Repressive Complex 2) or by directly influencing epigenetic modifications that silence tumor suppressor genes. Beyond transcriptional control, lncRNAs modulate key signaling pathways involved in cancer, including the PI3K/AKT/mTOR, Wnt/β-catenin, NF-κB, and TGF-β pathways, which play crucial roles in tumor proliferation, angiogenesis, epithelial-to-mesenchymal transition (EMT), and immune evasion [39–42]. Furthermore, some oncogenic lncRNAs are implicated in autophagy regulation, allowing cancer cells to adapt to metabolic stress and evade apoptosis, enhancing chemoresistance and tumor progression. For instance, SNHG6 (Small Nucleolar RNA Host Gene 6) acts as the competing endogenous RNA by sponging miR-204-5p, leading to E2F1 upregulation and cell cycle progression [41]. Additionally, CCAT2 (Colon Cancer-Associated Transcript 2) inhibits miR-145, further promoting HCC proliferation and metastasis [43]. Also, the HCV core protein influences carcinogenic progression and regulates the expression of TGFBRAP1 and HOTTIP by modulating miR-122 and miR-204 [44]. TUG1 (Taurine upregulated gene 1) is another lncRNA known to foster HCC progression via regulating various pathways. For eg: it activates JAK2/STAT3 pathway to promote proliferation in HCC via its interaction with miR-144 [45]. In addition, it acts as ceRNA for miR-132 for the regulation of the hedgehog pathway [46]. TUG1 also epigenetically silences KLF2, which further promotes cell growth and apoptosis, also linked to the autophagy-related axis involving the JAK-STAT pathway [47]. In HCC, elevated levels of lncRNA HOMER3-AS1 have been linked to enhanced tumor growth, migration, and invasion, contributing to poor patient survival. It is interesting that HOMER3-AS1 also plays a key role in M2 macrophage recruitment and polarization, further promoting cancer cell proliferation and tumor progression [48]. A recent study by Liu et al, 2024 reported that ST8SIA6-AS1 is an oncogenic lncRNA in HCC and uncovered its myc-dependent upregulation, providing a molecular basis for its role in carcinogenesis [49]. Another study identified the pseudogene-derived lncRNA viz. LOC344887 modulates the SHP1-regulated STAT3/HMGA2 signaling axis to cause the HCC progression [50]. ZNFX1 Antisense RNA 1 (ZFAS1) functions as an oncogenic lncRNA across various cancer types and has recently been known for its role in suppressing ferroptosis by regulating the miR-150/AIFM2 axis in HCC [51]. In addition, SH3BP5-AS1, recently identified as an oncogenic lncRNA in HCC, strongly correlated with advanced tumor stage. Its overexpression suppressed miR-6838-5p, leading to PTPN4 upregulation, which enhanced cell proliferation, invasion, and migration. Additionally, SH3BP5-AS1 promoted glycolysis via miR-6838-5p sponging and PTPN4 activation which is a non-receptor tyrosine phosphatase known to interact with autophagy regulators, and its upregulation contributes to autophagy activation, which in turn supports tumor cell survival and resistance under metabolic stress. Notably, HIF-1α directly regulated SH3BP5-AS1 transcription, further driving tumor progression [52].

**Fig. 2:**
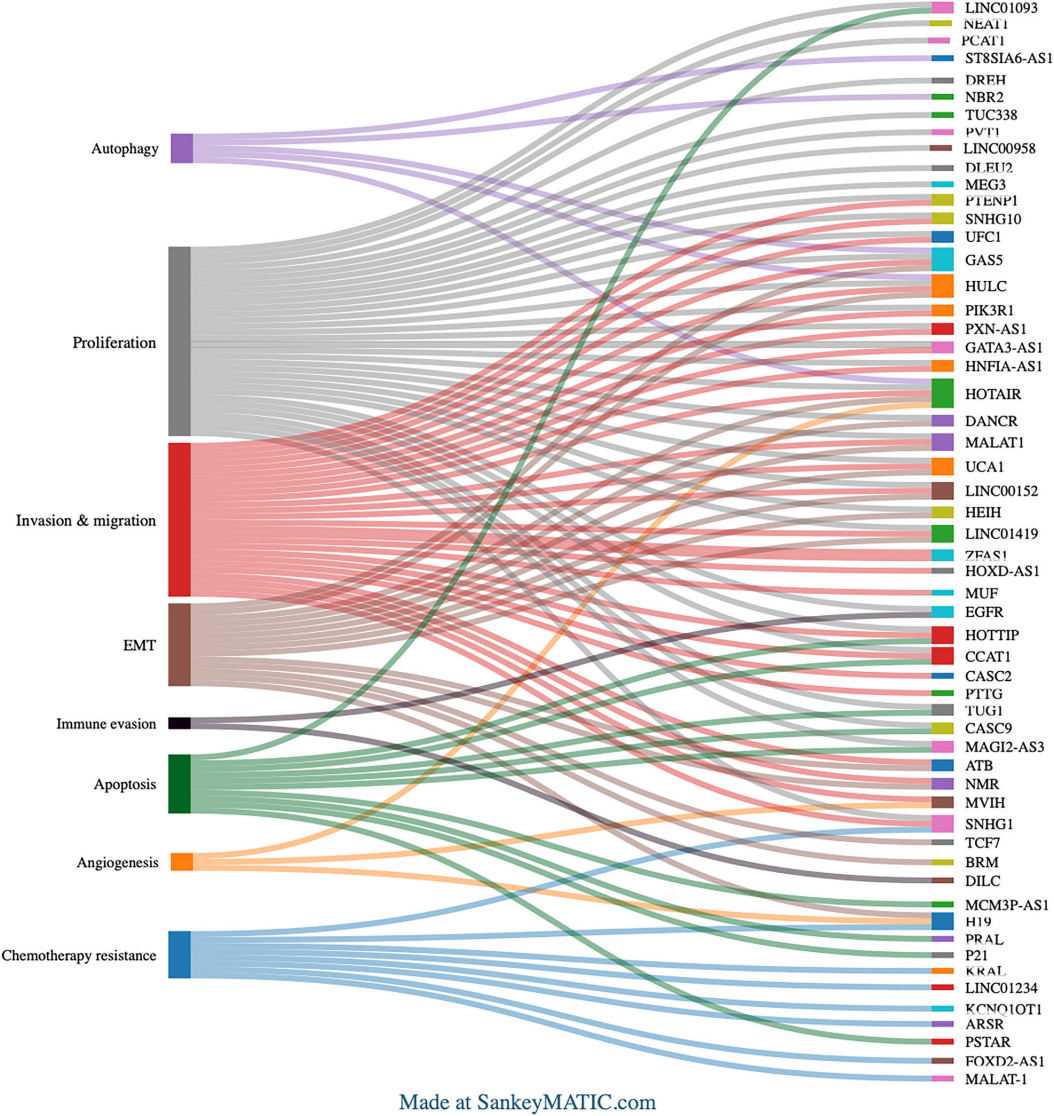
A Sankey diagram illustrating the complexity of functional roles of lncRNAs in HCC: the left panel represents key hallmarks of HCC. The right panel lists individual lncRNAs, each represented by a unique color. Connecting flows depict the association between specific lncRNAs and their respective cancer hallmarks. Some lncRNAs are involved in multiple processes, highlighting their multifunctional roles in HCC pathogenesis.

In addition to the role of lncRNAs as oncogenes, they also act as tumor suppressors. Tumor-suppressive lncRNAs act by inhibiting tumor proliferation, invasion, migration, epithelial-to-mesenchymal transition (EMT), and drug resistance, primarily by regulating apoptosis, cell cycle arrest, DNA repair, and autophagy. For instance, PTENP1 (phosphatase and tensin homolog pseudogene 1) is known to be downregulated in HCC, and overexpression of PTENP1 promotes autophagy and suppresses migration and invasion via interaction with the miR-193a-3p [53]. In HCC cells, lncRNA MAGI2-AS3 interacts with KDM1A, facilitating its recruitment to the RACGAP1 promoter, suppressing RACGAP1 expression through H3K4me2 demethylation [54]. Previous studies have demonstrated that lncRNA NBR2 acts as a tumor suppressor in HCC by inhibiting cytoprotective autophagy, thereby suppressing tumor growth. NBR2 downregulates Beclin1-dependent autophagy through the ERK and JNK pathways, highlighting its potential as a therapeutic target in HCC [55]. The liver-specific lncRNA LINC01093 suppresses HCC progression by interacting with IGF2BP1, promoting the decay of GLI1 mRNA, thereby inhibiting tumor growth; also acts as the significant prognostic marker in HCC patients [56]. In addition, Lnc-PIK3R1, transcriptionally repressed by YY1, suppresses HCC progression through the Lnc-PIK3R1/miR-1286/GSK3β axis, highlighting its tumor-suppressive role [57]. The Sankey diagram depicts the lncRNAs acting as oncogenes and tumor suppressors along with their role in regulating cancer hallmarks (Fig. 2).

### LncRNAs as biomarkers in HCC

Biomarkers play a vital role in early diagnosis, prognosis, and treatment monitoring of diseases like cancer, including HCC. LncRNAs have emerged as the potential non-invasive markers detected in tissue, plasma, serum, and urine, offering valuable diagnostic and prognostic insights. For instance, Wang et al., reported that GAS5 is downregulated in HCC and is related to tumor progression and poor prognosis [58]. MALAT1 is a widely studied lncRNA reported in various cancers, including its higher levels in HCC, which are associated with the recurrence and poor prognosis post-liver resection [59]. In addition, LINC01224 & SNHG15 are the two oncogenic lncRNAs related to histological grade, TNM staging, and vascular invasion [60, 61]. Also, HOXB-AS1, HOTAIR, TUG1 & CASC9 are overexpressed in HCC, promote proliferation, metastasis and are correlated with poor survival and advanced tumor staging, showing their utility as potential prognostic markers in HCC [62–64]. Apart from all these, several newly identified lncRNAs (CSMD1-1, HOXA-AS2, UC001kfo, PTTG3P, PDIA3P1) regulate myc signaling, PI3K/AKT pathway, and cancer stemness, influencing tumor aggressiveness and metastasis which can be further employed as the biomarkers in HCC [65–67]. Altogether, this suggests that lncRNAs hold great potential as diagnostic, prognostic, and therapeutic markers in HCC. Their dysregulated expression correlates with tumor stage, metastasis, and survival rates, making them promising candidates for non-invasive cancer detection and targeted therapy.

### LncRNAs mediating chemotherapy resistance

Apart from the role of lncRNAs as the oncogenes or the tumor suppressors, lncRNAs play a crucial role in inducing therapy resistance in HCC by influencing key cellular pathways such as drug metabolism, apoptosis, autophagy, and epithelial-to-mesenchymal transition. They can function as sensitizers or resistance mediators to various therapeutic agents, including chemotherapy, targeted therapy, and immunotherapy. Sorafenib, oxaliplatin, 5-flourouracil, cisplatin, etc. are the most commonly used chemotherapy drugs in HCC. Previous studies depict that the SNHG (small nucleolar RNA host gene) family has a role in the sorafenib resistance in HCC. For instance, SNHG1 induces sorafenib resistance by affecting the Akt pathway. A recent study by Shi et al. demonstrates that a well-studied lncRNA MALAT1 is dysregulated in the sorafenib-resistant HCC cells and its epigenetic modification viz. 5-methylcytosine methylation is responsible for the same via ELAVL1/SLC7A11 mediated ferroptosis in addition to the role of MALAT1 in sorafenib resistance via miR-140-5p pathway [68]. Similarly, epigenetic modification i.e. modification of N6 methyladenosine of lncRNA KIF9- AS1 promotes sorafenib resistance via upregulating SHOX2 expression [69]. On the other hand, lncTSI counteracts sorafenib resistance by suppressing miR-4726-5p and enhancing KCNMA1 expression in HCC [70]. Another study depicts that the downregulation of the lncRNA MBNL1-AS1 increases tripterine sensitivity by miR-708-5p mediated glycolysis [71]. Various lincRNAs also play a crucial role in modulating chemotherapy resistance. For example, LincROR targets the AP-2α/Wnt/ β catenin axis, driving adriamycin resistance [72]. In addition to this, both LINC01056 and LINC01089 also contribute to the sorafenib resistance in HCC [73, 74]. The long non-coding RNA LINC01532 contributes to lenvatinib resistance in hepatocellular carcinoma by preserving redox homeostasis [75]. Other well-studied lncRNAs and their relation with the chemotherapy drugs are depicted in Table 1.

**Table 1:** LncRNAs involved in chemotherapy drug resistance with regulatory mechanisms.

LncRNA	Mechanism	Chemotherapy drug resistance	Reference
KCNQ1OT1	Activates miR-7-5p	Oxaliplatin	[122]
HOTAIR	Disrupts miR-145-5p/HK2 Axis	Sorafenib	[123]
KRAL	miR-141/keap1/Nrf2 pathway	5-fluorouracil	[124]
PLAG1	PVT1/miR-195-5p axis	Sorafenib	[125]
Linc01234	miR-31-5p/MAGEA3 pathway	cisplatin	[126]
MALAT1	via miR-140-5p	sorafenib	[127]
DUBR	E2F1-CIP2A feedback	Oxaliplatin	[128]
FOXD2-AS1	via miR-140-5p	sorafenib	[129]
SNHG1	AKT pathway	sorafenib	[40]
ARSR	AKT pathway	doxorubicin	[130]
H19	miR-193-3p/PSEN1 pathway	5-fluorouracil	[131]
HULC	Stabilizing SIRT1	oxaliplatin	[88]

### LncRNAs regulate autophagy-related components

LncRNAs play a crucial role in modulating autophagy-related components in HCC by influencing autophagy initiation, autophagosome formation, lysosomal degradation, and other autophagy-related signaling pathways, ultimately affecting tumor growth and proliferation, survival, and drug resistance. MALAT1, H19, MEG3, NEAT1, GAS5, HOTAIR, HULC, SNHG1, and SNHG11 are some of the lncRNAs that regulate the autophagy components in different cancers (summarized in Table 2). In addition to these lncRNAs, a recent study reported the role of MIR210HG which is significantly increased in HCC and regulates cell proliferation by autophagy modulation [76]. Similarly, lncRNA ameliorates esophageal squamous cell carcinoma by promoting autophagy via the activation of the p53 pathway [77]. HOXA11-AS1 is another lncRNA that elevates autophagy along with the inhibition of apoptosis in acute T lymphoblastic leukemia by activating the miR-214-3p/ATG12 axis [78]. Not only this, autophagy in triple-negative breast cancer cells is also governed, in part, by the lncRNA RMST through its interaction with the miR-4295/ITPR1 pathway, highlighting a pivotal regulatory mechanism [79]. A recent study by Fu et al., reported that the sequesteration of miR-338-3p resulted in the activation of ATG12, and inhibited HCC progression [80]. LncRNA HITT, which has been implicated in various cancers, has recently been discovered to regulate autophagy by disrupting the formation of the ATG12–ATG5–ATG16L1 complex [81]. By promoting autophagy, LINC01106 also contributes to the malignant progression of lung adenocarcinoma [82]. Moreover, lncSNHG16 is linked to enhanced STAT3 signaling and concurrent inhibition of autophagy-mediated apoptosis [83]. By modulating the expression or activity of autophagy-related genes and interacting with critical signaling pathways—such as the AMPK/mTOR, PI3K/AKT, and Beclin1 pathways—lncRNAs orchestrate the dynamic balance between autophagy and tumor cell fate. Their regulatory influence extends to determining cellular responses to metabolic stress, therapeutic pressure, and hypoxic conditions, thereby impacting tumor proliferation, survival, invasion, and resistance to chemotherapy or targeted agents like sorafenib and lenvatinib.

**Table 2:** LncRNAs regulating the autophagy-related mechanism and affecting biological processes in various tumors.

LncRNA	Expression level	Tumor type	Autophagy- related mechanism	Biological process	Reference
MALAT1	Overexpression	Colorectal cancer	Increase in LC-3II/LC-3I	Increased proliferation, decreased apoptosis	[132]
	Overexpression	Gastric cancer	Increase in LC-3II/LC-3I via miR-204	Increased proliferation, increased cisplatin resistance	[133]
	Overexpression	Glioma	Decreased p62 and Increase in LC-3II/LC-3I via miR-101-3p	Increased proliferation	[134]
	Overexpression	Multiple myeloma	Increases Beclin-1 and LC3b	Increased proliferation and decreased apoptosis	[135]
	Overexpression	Pancreatic cancer	Increased LC3b & LAMP2, decreases p62	Increases MMP-3	[136]
CASC2	Underexpression	Colon cancer	Increases Beclin1 and LC3b	Increases Bax and decreases proliferation	[137]
	Overexpression	Glioma	Decreases Beclin1 and LC3b	Decreases migration and increases mTOR	[138]
H19	Underexpression	Breast cancer	Decreases Beclin1 and LC3b	Decreased tamoxifen resistance	[139]
	Overexpression	Colorectal cancer	Increased LC3b & decreased p62	Increased proliferation and chemoresistance	[115]
	Overexpression	Glioma	Decreased autophagy	Increased proliferation and migration	[140]
	Underexpression	Hepatocellular carcinoma	Decreased LC3b & Beclin1	Increased proliferation and decreased caspase-9	[141]
MEG3	Underexpression	Bladder cancer	Increased LC3b	Decreased apoptosis	[142]
	Overexpression	Hepatocellular carcinoma	Decreased Beclin1 and LC3b	Decreased proliferation	[84]
NEAT1	Underexpression	Colorectal cancer	Decreased Beclin1 and LC3b	Increased chemosensitivity	[143]
	Overexpression	Hepatocellular carcinoma	Increased LC3b and ATG3	Decreased sorafenib-induced growth inhibition	[86]
GAS5	Overexpression	Breast cancer	Increased Beclin1 and LC3b	Increased chemosensitivity	[144]
	Overexpression	Glioma	Decreased LC3b and increased p62	Decreased proliferation and increased chemosensitivity	[145]
SNHG1	Underexpression	Prostate cancer	Increased LC3b and Beclin1		[146]
	Underexpression	Renal cell carcinoma	Decreased LC3b and Beclin1	Decreased proliferation and increased apoptosis	[147]
SNHG11	Underexpression	Hepatocellular carcinoma	Decreased LC3b and Beclin1	Decreased migration and invasion	[148]
	Underexpression	Gastric cancer	Decreased LC3b	Increased caspase-3	[149]
HOTAIR	overexpression	Hepatocellular carcinoma	Increased LC3b and ATG3	Increased proliferation	[150]
HULC	Overexpression	Gastric cancer	Increased LC3b and Beclin1	Increased cisplatin resistance	[151]
	Overexpression	Hepatocellular carcinoma	Increased LC3b	Increased proliferation	[89]
	Underexpression	Prostate cancer	Increased LC3b	Increased irradiation sensitivity	[152]
DANCR	Overexpression	Acute myeloid leukemia	Increased LC3b	Increased cytarabine resistance	[153]
	Underexpression	Breast cancer	Increased LC3b & ATG5	Increased caspase-3 and caspase-9	[154]
	Underexpression	Gastric cancer	Increased LC3b and Beclin1	Increased apoptosis	[155]
	Underexpression	Hepatocellular carcinoma	Decreased autophagy	Decreased proliferation	[94]

## LncRNAs as the master regulators of autophagy: implications for hepatocellular carcinoma

**Fig. 3:**
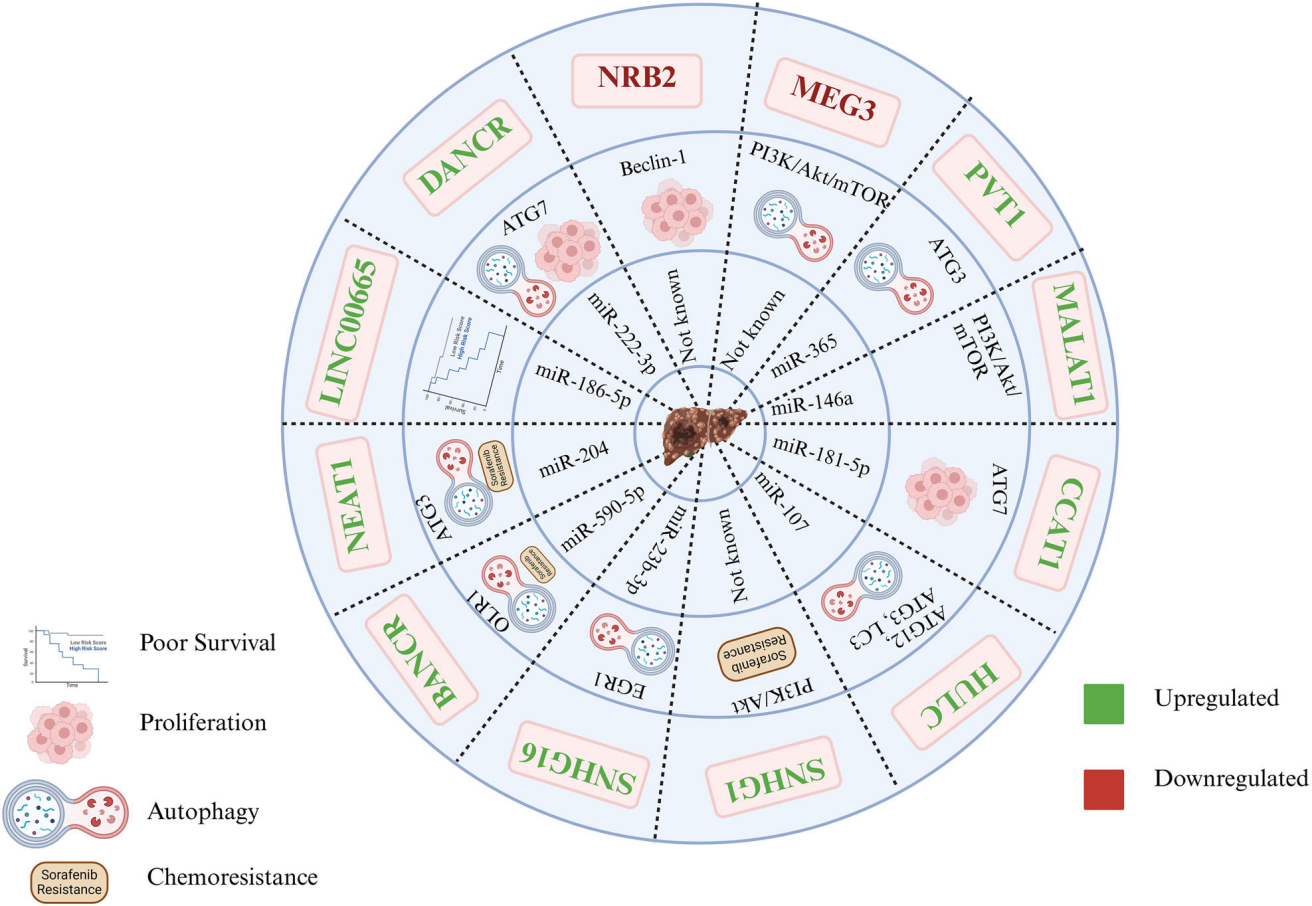
Schematic representation of lncRNA-miRNA interactions and their mechanistic contributions to HCC progression and therapy resistance. Each section of the circular plot highlights a specific lncRNA and its downstream molecular interactions or targets. Outer ring: upregulated lncRNA in green and downregulated lncRNA in red, while inner layers show the miRNAs regulated by lncRNAs and molecular pathways or target genes involved.

The interplay between the lncRNAs and autophagy significantly impacts the HCC pathogenesis, influencing tumor progression, metastasis, and therapy resistance. In addition, the differential expression of lncRNAs highlights their diverse and context-dependent role, which include valuable biomarkers for predicting survival outcomes and recurrence rates. In this context, overexpression of MEG3 inactivates Beclin-1 signaling to inhibit autophagy and activates the PI3K/Akt/mTOR pathway [84, 85]. On the other hand, NBR2, a long intergenic ncRNA known to be downregulated in HCC, induces Beclin-1-dependent autophagy, resulting in increased cell proliferation [55]. Moreover, some other lncRNAs that are known to be upregulated in HCC include NEAT1, SNHG16, HULC, MALAT1, CCAT1, PVT1, LINC00665, DANCR, SNHG1, and BANCR. Most of these lncRNAs act by spongingthe miRNAs, which relieves the inhibition caused by the miRNAs on the protein target. The Cancer Genome Atlas identified NEAT1 upregulation in HCC, with its negative correlation with the survival rates. It also acts as a sponge for the miR-204, decreasing the levels and causing further activation of ATG3 to induce autophagy and compromise sorafenib efficacy as well [86]. Another lncRNA, SNHG16, inhibits miR-23b-3p via the upregulation of EGR1 to promote autophagy and suppress apoptosis [87]. HULC triggers the autophagy in HCC via various mechanisms, viz. inhibiting the interference of miR-107 with ATG12, by decreasing the levels of miR15a thereby enhancing the interplay between LC3 and ATG3, and lastly by the stabilization of Sirt1 [88–90]. A well-explored lncRNA, MALAT1, is overexpressed in HCC, and it targets miR-146a, further preventing its binding to the 3’UTR of PI3K [39]. Similarly, CCAT1 sponges the miR-181a-5p to promote autophagy by regulating ATG7 and is known to promote cell proliferation in HCC [91]. PVT1, an oncogene in multiple cancers, acts as a miR-365 sponge to prevent its binding to the 3’UTR of ATG3, facilitating autophagy in HCC cells [92]. LINC00665 and MAP4K3 LncRNAs positively correlate with HCC and negatively correlate with miR-186-5p. Depletion of this lincRNA causes autophagy promotion, while the elevated LINC00665 observed in HCC tissues is linked with shorter overall survival in patients [93]. LncRNA DANCR enhances the ATG7 expression by sponging miR-222-3p, facilitating HCC proliferation and autophagy [94]. Conversely, BANCR suppresses miR-590-5p expression, which then targets and reduces the expression of OLR1, activating autophagy and sorafenib-induced chemoresistance [95]. In addition to this, SNHG1 promotes sorafenib resistance through the activation of the Akt pathway. Although lncRNAs exert their effects through various mechanisms like gene expression regulation, post-transcriptional regulation, epigenetic regulation, protein interaction, and miRNA sponging, the mechanistic studies of lncRNAs in regulating the autophagy highlight its differential expression and interactions with miRNAs as their potential to act as the biomarkers and therapeutic targets. Targeting lncRNA-mediated pathways could offer promising strategies to overcome drug resistance and improve patient outcomes. The autophagy-mediated lncRNAs regulating HCC via different pathways are summarised in Fig. 3.

## Therapeutic targeting of lncRNA-autophagy axis in HCC

### Pharmacological modulation of autophagy

The pharmacological targeting of autophagy has garnered significant attention in cancer, neurodegeneration, and metabolic diseases. Pharmacological targeting of autophagy includes the usage of autophagy inducers, which can be mTOR inhibitors, AMPK activators, sirtuin activators, or lysosome-targeting agents, whereas autophagy inhibitors are the lysosomal inhibitors, autophagosome formation inhibitors, or PI3K and ATG inhibitors. mTOR inhibitors, derived from Rapamycin, are known as Rapamycin analogs or Rapalogs, which include Everolimus (RAD001), Deforolimus (AP23573), and Temsirolimus (CCI-779). For instance, oral administration of RAD001 in mice with patient-derived HCC xenografts leads to a dose-dependent inhibition of tumor growth [96]. A Phase III trial of Everolimus in HCC was terminated early due to slow enrollment of patients and high screen failure rates (NCT01379521), whereas Deforolimus and Temsirolimus have shown no significant trials in HCC. Similarly, treatment with Rapamycin and CCI-779 in PLC/PRF/5 human HCC cells induced G1-phase cell cycle arrest, reducing tumor growth in both colony formation assays and xenograft models [97]. Furthermore, long-term RAD001 treatment significantly delayed DNA damage-induced liver tumor development, highlighting its potential as a therapeutic strategy for HCC [98]. AMPK activators stimulate autophagy by inhibiting mTOR and activating the ULK1. Metformin is the most widely used anti-diabetic drug used to induce autophagy by activating AMPK. In addition, metformin is also known to activate autophagy and apoptosis via AMPK-independent pathways, collectively preventing cancer development [99]. A combination of metformin and sorafenib has completed Phase II clinical trial but not yet approved for the HCC treatment (NCT02672488). Preclinical studies show that Resveratrol, a potent sirtuin-1 activator, is a well-known anticancer drug, and it is reported to induce mitophagy through the MALAT1/miR-143-3p/RRM2 axis, effectively inhibiting cancer progression in hepatocellular carcinoma [100]. In addition to this, lysosome-targeting agents act either by enhancing the lysosomal function to promote the autophagic flux, for example trehalose (enhances lysosomal clearance) as shown by preclinical studies, or by inhibiting the lysosomal function, blocking autophagosome-lysosome fusion for eg: chloroquine (increases lysosomal pH), and bafilomycin A1 (V-ATPase inhibitor, prevents acidification) [101, 102]. In addition, combining lysosome-targeting compounds, such as VP, with approved chemotherapeutic agents presents a promising strategy to overcome chemoinsensitivity in HCC by counteracting passive lysosomal sequestration of anticancer drugs [103]. In addition, combination of CQ/HCQ with chemotherapy has completed Phase I clinical trials in advanced solid tumors which include HCC as well (NCT02071537). Apart from these, widely used modulators of autophagy, such as mTOR inhibitors, AMPK activators, and lysosome-targeting agents, some inhibitors of the autophagy pathway, which include PI3K inhibitors and ATG inhibitors, are also being used to modulate autophagy in HCC. Wortmannin is a widely used PI3K inhibitor [104]. Also, tissue factor knockdown in HCC cells enhances autophagy and reduces cell survival, which are reversed by autophagy inhibitors 3-MA or Spautin-1 (a Beclin-1 specific inhibitor) [105]. However, PI3K inhibitors and Beclin-1 pathway inhibitors show only preclinical evidence. These pharmacological modulators of autophagy offer promising therapeutic strategies for HCC, either by enhancing autophagic flux to promote cancer cell death or by inhibiting autophagy to prevent tumor survival under stress conditions. Further research and clinical trials are essential to optimize these approaches and develop effective combination therapies for improved cancer treatment outcomes.

### LncRNAs in autophagy-mediated HCC: Biomarker and Therapy prospects

Biomarkers represent a transformative avenue in advancing disease diagnosis, treatment, and prevention, thereby enhancing patient outcomes and deepening our understanding of complex diseases. The identification of biomarkers has been revolutionized by the state-of-art methodologies, including proteomics, transcriptomics, whole genome sequencing, and array-based techniques. Beyond conventional tissue and serum biomarkers, non-coding RNAs, especially miRNAs and lncRNAs have emerged as the pivotal diagnostic and prognostic tools in HCC. Apart from this, autophagy-related lncRNAs not only provide insights into the molecular mechanisms of HCC but also offer promising opportunities for novel biomarkers and therapeutic strategies in HCC management. However, these are not explored to a deeper extent yet. In this context, a bioinformatics study showed that the risk prediction model constructed using the panel of autophagy-related lncRNAs (PRRT3- AS1, RP11-479G22.8, RP11-73M18.8, LINC01138, CTD-2510F5.4, RP11-324I22.4, and CTC-297N7.9) had the strong predictive capability and holds strong promise, which depicts their robust potential as the prognostic and diagnostic biomarkers for HCC [106]. Another study using bioinformatic approaches also depicted that five autophagy-related lncRNAs (TMCC1-AS1, PLBD1-AS1, MKLN1-AS, LINC01063, and CYTOR) demonstrated stratification of high- and low-risk HCC patients. Moreover, Dent et al. also identified a signature having potential to predict therapeutic outcomes in HCC patients, including responses to chemotherapy and immunotherapy [107]. Wang et al. showed the association of the survival of HCC patients with the immuno-autophagy-related lncRNAs which includes the panel of ten lncRNAs (BACE1-AS, MIR210HG, AC073896.4, AC099850.3, AC026401.3, MAPKAPK5-AS1, LINC01018, CYTOR, AC115619.1, and F11-AS1). The distinct roles of these lncRNAs, including their potential contributions to tumor growth and proliferation, cannot be overlooked [108]. Also, low NBR2 (a tumor suppressor) levels related to worse survival and increased malignancy in HCC, thus acting as the potential biomarker [55]. In addition to this, HULC (an oncogenic lncRNA) serves as a valuable prognostic biomarker and facilitates the HCC progression while also representing a therapeutic target for this malignancy [109]. Nevertheless, it is worth mentioning that the heterogeneity in the clinical samples submitted to repositories, coupled with the use of diverse computational analytical methods, significantly limit the effectiveness of these predictive markers in clinical settings. In addition to the prognostic and diagnostic significance of lncRNAs, recent studies have highlighted the significant role of lncRNAs in predicting the reoccurrence post-surgery in HCC patients. For instance, a study by Wu et al. showed that the increased levels of HOTTIP are an independent factor for the recurrence after liver transplantation [110]. In addition, MALAT1 and HOTAIR are oncogenic lncRNAs associated with the increased risk of early recurrence in HCC patients undergoing surgical resection [111, 112]. Recent literature has also shown the vital role of lncRNAs in sorafenib resistance, which is the first-line treatment for the molecular targeted therapy in HCC. For example, inhibition of SNHG1 represses Akt signaling pathway while enhancing apoptosis and autophagy, thereby mitigating the sorafenib resistance [40]. SNHG16 contributes to sorafenib resistance by downregulating miR-140-5p, a miRNA that promotes cancer migration and invasion [113]. Two studies have demonstrated the role of NEAT1 in conferring the sorafenib resistance via NEAT1/miR-335/c-met axis and NEAT1miR-204/ATG3 axis [86, 114]. Altogether, this depicts that lncRNAs have emerged as pivotal players in HCC, offering immense potential as diagnostic, prognostic, and therapeutic biomarkers.

## Conclusive remarks

In conclusion, lncRNAs represent a transformative frontier in HCC management, potentially revolutionizing current diagnostic and therapeutic paradigms. Continued exploration of their regulatory roles in autophagy and tumor biology will pave the way for innovative, lncRNA-based strategies to combat this aggressive malignancy. However, recent findings suggest strategies like ASOs (antisense oligonucleotides), siRNAs, and CRISPRs for targeting of the autophagy-related lncRNAs in cancer. Several studies have demonstrated the feasibility of using ASOs and siRNAs to silence oncogenic lncRNAs involved in autophagy regulation. For an instance, Wang et al. demonstrated knockdown of lncRNA HOTAIR using siRNA inhibited autophagy and sensitized colorectal cancer cells to chemotherapy [115]. Similarly, Liu et al. demonstrated that suppression of H19 using siRNA impaired autophagic flux and reduced tumor cell survival in CRC [116]. Both H19 and HOTAIR are also overexpressed in HCC and implicated in autophagy regulation, suggesting that siRNA-mediated knockdown strategies tested in CRC may be translatable to HCC. In addition, there is increasing clinical interest in siRNA-based formulations. Notably, patisiran (Onpattro) and givosiran (GIVLAARI), the FDA-approved siRNA therapeutics, have validated the clinical feasibility of siRNA delivery using lipid nanoparticles [117]. Moreover, ongoing trials in oncology are evaluating siRNA drugs such as TKM-080301, siG12D-LODER, and STP705, highlighting the therapeutic potential of this class [118–120]. These advancements suggest that autophagy-regulating lncRNAs in HCC could be targeted using similar nanoparticle-based siRNA delivery systems. Emerging studies have employed CRISPR/Cas9 to delete lncRNAs implicated in autophagy and drug resistance. For instance, targeted silencing of lncRNA-GACAT3 using CRISPR-Cas13 technology inhibits proliferation and migration, and induces apoptosis in bladder cancer cells by enhancing the expression of p21, Bax, and E-cadherin [121]. Many autophagy-related lncRNAs, including *H19*, *HOTAIR*, *PVT1*, and *SNHG16*, are highly expressed in HCC and correlate with poor prognosis, chemoresistance, and enhanced autophagic activity. Given their dual role in promoting autophagy and tumor survival, these lncRNAs are compelling candidates for targeted inhibition using nucleic acid-based therapeutics. Despite the promising findings, several challenges remain. The heterogeneity of clinical samples, variability in computational methodologies, and limited mechanistic studies hinder the translation of lncRNA research into clinical applications. Future studies must address these limitations by employing robust experimental designs, standardized analytical approaches, and comprehensive validation in diverse patient cohorts.

## Future outlook and challenges

**Fig. 4:**
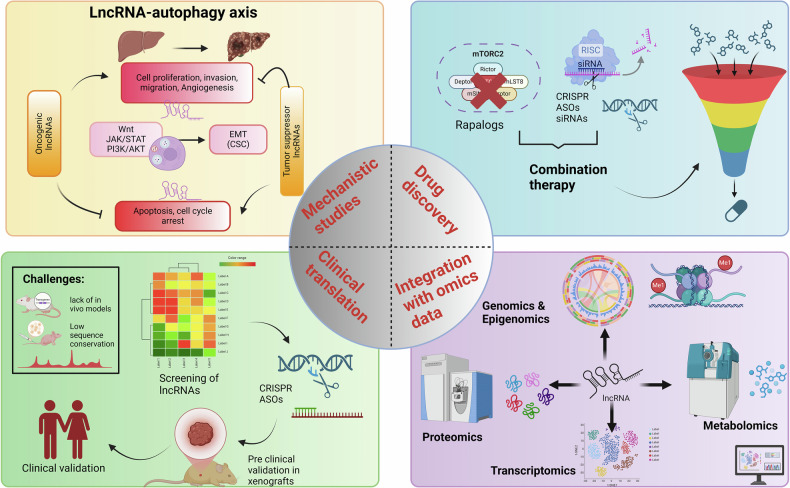
Unraveling Mechanisms and Advancing Therapeutic Frontiers in lncRNAs and Autophagy crosstalk in HCC. Intricate interplay between lncRNAs and autophagy in HCC progression and therapeutic targeting: the wheel highlights four critical future directions: mechanistic studies, drug discovery, integration with omics data, and clinical translation, emphasizing the need for a multidisciplinary approach to leverage lncRNAs as potential therapeutic targets in HCC.

Hepatocellular carcinoma (HCC) remains a leading cause of cancer-related mortality, with rising incidence and limited treatment options. Identifying novel molecular targets and therapeutic strategies is crucial, given its highly aggressive nature and poor prognosis. Emerging evidence highlights the intricate interplay between long non-coding RNAs (lncRNAs) and autophagy, which significantly contribute to HCC initiation, progression, metastasis, and therapeutic resistance. Given their multifaceted role in cancer biology, oncogenic lncRNAs are now being explored as potential therapeutic targets. The crosstalk between lncRNAs and autophagy offers a promising avenue for therapeutic intervention. Strategies aimed at restoring tumor-suppressive lncRNAs or inhibiting oncogenic lncRNAs, in combination with autophagy modulators, hold the potential for improving treatment efficacy and overcoming drug resistance in HCC. Despite significant advancements in understanding their molecular functions, several challenges remain before lncRNA-autophagy interactions can be leveraged for clinical applications. Foremost, most of the studies focus on changes in the lncRNA expression in HCC rather than validating their direct functional roles in autophagy regulation. So, more studies are required on the gain and loss of function. This is also attributed to the limited in vivo models as only a few studies depict the usage of in vivo models (such as genetically engineered mouse models (GEMMs) or patient-derived xenografts (PDXs) for lncRNA-autophagy interactions. Secondly, many lncRNAs exhibit low sequence conservation, making it difficult to develop relevant animal models for studying their function in autophagy and HCC progression. Third andmost crucial, mostly studies remain in the preclinical stage, and high-throughput screening technologies (CRISPR, RNA-seq, single-cell sequencing) are needed to identify clinically relevant lncRNAs regulating autophagy. Although autophagy inhibitors/inducers and lncRNA-targeting approaches (siRNAs, ASOs, CRISPR) have shown promise, there are no clinically approved lncRNA-based therapies for HCC (Fig. 4). In future:

1. Comprehensive multi-omics approaches integrating transcriptomics, epigenomics, and proteomics are needed to map the lncRNA-autophagy signaling axis in HCC.
2. Circulating lncRNA regulating the autophagy can act as the non-invasive biomarkers for the early detection of HCC.
3. Developing lncRNA-based therapeutics (e.g., ASOs, siRNAs, small-molecule inhibitors) combined with autophagy modulators may improve HCC treatment outcomes.
4. Investigating how lncRNA-mediated autophagy affects hypoxia response, glycolysis, immune evasion, and resistance to chemotherapy/targeted therapy in tumor microenvironment will provide new therapeutic insights.

While lncRNA-autophagy interactions represent a promising frontier in HCC research, overcoming existing challenges in mechanistic validation, therapeutic targeting, and clinical translation will be crucial (Fig. 4). Future studies integrating advanced gene-editing tools, functional genomics, and precision medicine approaches could unlock the full therapeutic potential of lncRNA-autophagy regulation in HCC treatment.

## Data Availability

The data that support these findings is available with the corresponding author, and enquiries should be directed to the authors of the manuscript.
